# Cardiovascular disease recurrence and long-term mortality in a tri-ethnic British cohort

**DOI:** 10.1136/heartjnl-2020-317641

**Published:** 2020-10-16

**Authors:** Manav V Vyas, Nish Chaturvedi, Alun D Hughes, Michael Marmot, Therese Tillin

**Affiliations:** 1 Division of Neurology, University of Toronto, Toronto, Ontario, Canada; 2 MRC Unit for Lifelong Health and Ageing at UCL, University College London, London, UK; 3 Epidemiology and Public Health, University College London, London, UK

**Keywords:** epidemiology, quality and outcomes of care, cardiac risk factors and prevention

## Abstract

**Objective:**

Ethnic differences in cardiovascular disease incidence, but not cardiovascular disease recurrence, are reported. We characterised long-term risk of major adverse cardiovascular event (MACE) and mortality following a non-fatal cardiovascular event in a British cohort of South Asians, African Caribbeans and Europeans.

**Methods:**

We identified index and recurrent cardiovascular events and mortality between 1988 and 2017 using hospital records and death registry. Using multivariable hazards models, we separately calculated the adjusted HR of MACE and death following index event, adjusting for demographics, vascular and lifestyle risk factors. Using interaction terms, we evaluated if decade of index event modified the association between ethnicity and outcomes.

**Results:**

South Asians were younger at the index event (median age 66 years, n=396) than Europeans (69 years, n=335) and African Caribbeans (70 years, n=70). During 4228 person-years, of the 801 patients, 537 developed MACE and 338 died, with the highest crude rate of MACE in South Asians. On adjustment of baseline factors, compared with the Europeans, the higher risk of MACE (HR 0.97, 95% CI 0.77 to 1.21) and the lower risk of mortality (HR 0.95, 95% CI 0.72 to 1.26) in South Asians was eliminated. African Caribbeans had similar outcomes to Europeans (HR MACE 1.04, 95% CI 0.74 to 1.47; and HR death 1.07, 95% CI 0.70 to 1.64). Long-term survival following an index event improved in South Asians (p_trend_ 0.02) and African Caribbeans (p_trend_ 0.07) compared with Europeans.

**Conclusions:**

Baseline vascular risk factors explained the observed ethnic variation in cardiovascular disease recurrence and long-term mortality, with a relative improvement in survival of minority ethnic groups over time.

## Introduction

Previous studies have reported ethnic variation in the incidence of cardiovascular disease, one of the leading causes of death and disability worldwide.^1^ South Asian ethnicity has been associated with a greater risk compared with European ethnicity, despite adjusting for vascular and lifestyle risk factors, and partly due to a higher prevalence of diabetes.^2^ Whereas, people of African or Caribbean heritage may have similar risk of cardiovascular disease compared with their white counterparts, except for cerebrovascular events.^2^ The 2016 European guidelines and the 2019 American College of Cardiology/American Heart Association guidelines on cardiovascular disease prevention acknowledge ethnic differences in cardiovascular disease and recommend strategic efforts to reduce these differences.^3 4^

The current literature on ethnic differences in cardiovascular disease recurrence is sparse. South Asians and African Caribbeans have a higher prevalence of diabetes compared with other ethnic groups^5^ and diabetes increases the risk of cardiovascular disease recurrence.^6^ Our aim was to evaluate long-term risk of cardiovascular disease recurrence and mortality in three ethnic groups: South Asian (from the Indian subcontinent), African Caribbean (from West Africa or the Caribbean islands) and European, with the hypothesis that South Asians, but not African Caribbeans, will have a higher risk of cardiovascular disease recurrence compared with Europeans. We further hypothesised that diabetes modifies the association between ethnicity and cardiovascular event recurrence, and that ethnic differences in recurrence have attenuated over time.

## Methods

### Setting and population

The Southall And Brent REvisited (SABRE) cohort is a tri-ethnic cohort of people of European, South Asian and African Caribbean ethnicity residing in the London borough of Southall and Brent between 1998 and 1991.^7 8^ It included men and women aged 45–69 years identified from either primary care lists or from industrial workforce records.^9^

We linked cohort participants’ data to Hospital Episode Statistics (HES) and UK national death registry from the Office of National Statistics (ONS). The final follow-up date for which we had complete data for this analysis was 31 March 2017. We ascertained index cardiovascular event from the start of the cohort to 1 January 2015 (ascertainment period) if an eligible International Classification of Diseases (ICD)-9 or ICD-10 code for cardiovascular disease was listed in the *main* diagnostic fields of their hospital admission (online supplemental e-table 1) in the HES database.^10^ We defined an index composite cardiovascular event as: myocardial infarction (ICD-9 410–11, ICD-10 I21-22), ischaemic heart disease including angina (ICD-9 412–414, ICD-10 I20) or stroke or transient ischaemic attack (TIA) (ICD-9 430–431, 434, 436, 3623, ICD-10 I60-64, H340-341, G45 except G456 and G457). We excluded participants who did not have a cardiovascular event during the ascertainment period, and those who died within 30 days following the index cardiovascular event (fatal event). We also excluded those with a known history of cardiovascular disease at cohort inception (based on initial history and physical examination) because we did not know the time of their previous event.

10.1136/heartjnl-2020-317641.supp1 Supplementary data

### Exposure and outcomes

The exposure of interest was ethnicity which was agreed on between participants and a trained interviewer based on appearance and parental origin of the participants. We considered Europeans as the reference group. All South Asian and African Caribbean participants in this study were first-generation migrants to the UK.

The primary outcome of interest was a major adverse cardiovascular event (MACE). It was defined as a composite of cardiovascular event recurrence or death due to cardiovascular disease. Cardiovascular event recurrence was ascertained based on a previously validated definition of first-ever hospital admission where the *main diagnosis* of the hospitalisation was listed as either stroke, TIA, myocardial infarction or ischaemic heart disease (online supplemental e-table 1).^10^ If another event occurred during follow-up, only the first event was included. Death due to cardiovascular disease was ascertained based on the death certificate listing cardiovascular disease as one of the responsible causes of death. Among those who had a cardiovascular event recurrence, we obtained information on the date of hospital admission and that of the end of the hospital spell to ascertain the duration of hospitalisation.^11^

The secondary outcomes of interest were: cardiovascular event recurrence, and all-cause mortality. Cardiovascular event recurrence was in essence MACE except for death from cardiovascular disease. Information on the date of death and cause of death was obtained from the UK national death registry and from the ONS.

### Statistical analyses

We used a Cox proportional hazards model to calculate cause-specific HRs of MACE with death from non-cardiovascular causes as a competing event; accounting for the latter allows to produce unbiased estimates as it precludes the occurrence of the event of interest.^12^ We assigned the date of discharge from the index cardiovascular event as time zero. We censored individuals at the end of the follow-up in 31 March 2017, at the time of event of interest, or, at the time of departure for those who emigrated. We calculated unadjusted, age-adjusted and sex-adjusted, and multivariable adjusted HRs of MACE in South Asians and African Caribbeans compared with Europeans. Multivariable models included: age and duration of hospitalisation at the time of index event, sex, together with baseline variables including diabetes, hypertension, serum total triglycerides, body mass index, smoking status, alcohol intake, regular consumption of fruits and vegetables and physical activity (online supplemental e-tables 2 and 3 for details). While some of these covariates could be considered mediators in the association between ethnicity and cardiovascular disease recurrence, we selected them based on previous large-scale studies on the association between ethnicity and incidence of cardiovascular disease.^5^ We used similar methods to evaluate the association between ethnicity and non-fatal cardiovascular event recurrence, with death from any cause as a competing risk, and for all-cause mortality, we censored individuals at the end of follow-up.

Using prespecified subgroup analyses, we evaluated the association between ethnicity and all outcomes of interest based on the type of index event: coronary event (myocardial infarction or ischaemic heart disease) or cerebrovascular event (stroke or TIA). In the subgroup of those who had a coronary event, we further adjusted for receipt of coronary intervention at the index event (information on coronary bypass surgery or percutaneous coronary intervention obtained using Office of Population Censuses and Surveys Classification of Surgical Operations and Procedures codes) (online supplemental e-table 1).^13^

In multivariable adjusted models, we added an interaction term to evaluate the modifying effect of diabetes on the associations between ethnicity and MACE and all-cause mortality. In a sensitivity analyses, we evaluated the interaction between ethnicity and baseline serum fasting glucose (mmol/L) on MACE.

Using an interaction term in the multivariable adjusted model, we evaluated whether the adjusted hazard of MACE and all-cause mortality varied across ethnic groups based on the decade of the index cardiovascular event: before 2000 (1990), between 2000 and 2010 (2010) and in or after 2010 (2010). We calculated a p value for trend in the change over time.

We conducted analyses using SAS V.9.4 2002–2012 by SAS Institute, Cary, North Carolina, USA and Stata Statistical Software: release 14, College Station, Texas, USA.

## Results

**Figure 1:**
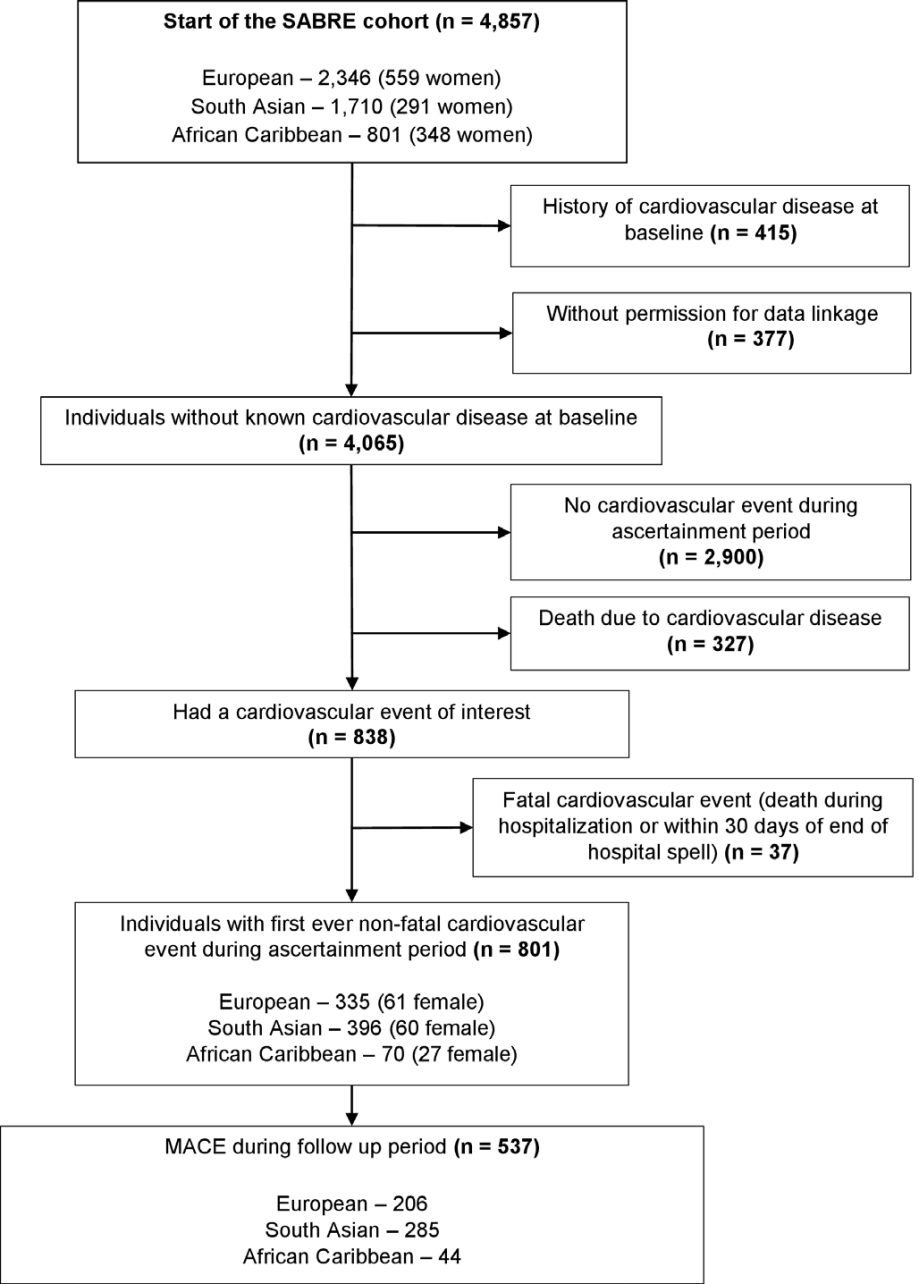
Cohort selection. Ascertainment period: between 1 June 1988 and 1 January 2015. Follow-up period: from index cardiovascular event to 31 March 2017, mean 5.3 years. MACE, major adverse cardiovascular event—composite of cerebrovascular event (stroke/transient ischaemic attack), coronary event (myocardial infarction/ischaemic heart disease) or death due to cardiovascular disease; SABRE, Southall And Brent REvisited.

The SABRE cohort consisted of Europeans (n=2059), South Asians (n=1517) and African Caribbeans (n=630), and had an initial response rate of 63%. Of these, we included 801 patients who had a non-fatal cardiovascular event during the ascertainment period, 674 (84.1%) coronary events and 127 (15.9%) cerebrovascular events (figure 1). Of those included, 335 (41.8%) were of European, 396 (49.5%) were of South Asian and 113 (8.7%) were of African Caribbean descent. South Asians were younger at the time of index cardiovascular event compared with Europeans (median age 66 vs 70 years) (table 1). Other baseline characteristics are described in table 1 and online supplemental e-table 2.

**Table 1:** Baseline characteristics of a tri-ethnic cohort of patients with an index non-fatal cardiovascular event in London, England

	European (n=335)		South Asian (n=396)		African Caribbean (n=70)	
Had MACE during follow-up	No	Yes	No	Yes	No	Yes
	128 (38.2)	207 (61.8)	110 (27.8)	286 (72.2)	26 (37.1)	44 (62.9)
Demographic information						
Median age (years) at index event	70 (65–76)	69 (63–75)	67 (63–71)	64 (58–71)	73 (65–76)	66 (63–74)
Female, n (%)	24 (18.8)	37 (17.9)	19 (17.3)	41 (14.3)	13 (50.0)	14 (31.8)
Median years of education	10 (10–11)	10 (9–11)	12 (10–15)	12 (10–14)	10 (9–11)	10 (9–11)
Vascular risk factors						
Known diabetes, n (%)	4 (3.1)	23 (11.1)	24 (21.8)	82 (28.7)	5 (19.2)	14 (31.8)
Known hypertension, n (%)	6 (4.7)	24 (11.6)	18 (16.4)	41 (14.3)	8 (30.8)	12 (27.3)
Median fasting blood glucose (mmol/L)	5.4 (5.1–5.7)	5.5 (5.1–6.0)	5.7 (5.1–6.3)	5.6 (5.2–6.5)	5.9 (5.3–6.4)	6.0 (5.3–6.8)
Median total cholesterol (mmol/L)	6.1 (5.4–6.9)	6.3 (5.6–7.1)	5.9 (5.2–6.4)	6.0 (5.4–6.8)	5.3 (4.7–6.1)	6.0 (5.0–6.9)
Median HDL (mmol/L)	1.3 (1.1–1.6)	1.2 (1.0–1.5)	1.2 (1.0–1.4)	1.1 (1.0–1.3)	1.7 (1.4–1.9)	1.4 (1.2–1.7)
Median triglycerides (mmol/L)	1.5 (1.1–1.9)	1.6 (1.1–2.3)	1.7 (1.2–2.2)	1.9 (1.3–2.9)	1.1 (0.8–1.5)	1.2 (1.0–1.8)
Median systolic BP (mm Hg)	122 (114–134)	126 (115–127)	122 (114–137)	126 (114–137)	128 (119–135)	131 (121–139)
Median diastolic BP (mm Hg)	79 (73–85)	79 (72–86)	80 (74–86)	81 (74–88)	80 (75–85)	84 (75–89)
Median BMI (kg/m^2^)	26.4 (23.6–28.7)	26.0 (24.0–29.0)	25.9 (24.0–29.0)	26.1 (24.3–28.3)	26.3 (24.4–29.7)	28.0 (25.3–29.8)
Median waist-to-hip ratio	0.93 (0.85–0.97)	0.94 (0.88–0.99)	0.97 (0.92–1.02)	0.97 (0.93–1.01)	0.88 (0.84–0.97)	0.94 (0.89–0.98)
Lifestyle factors						
Physically active, n (%)	78 (60.9)	115 (55.6)	44 (40.0)	125 (43.7)	13 (50.0)	22 (50.0)
Healthy diet, n (%)	50 (39.1)	67 (32.4)	43 (39.5)	99 (35.1)	13 (50.0)	15 (34.1)
Ex-smoker, n (%)	49 (38.3)	72 (34.8)	7 (6.4)	24 (8.4)	2 (7.7)	5 (11.4)
Current smoker, n (%)	36 (28.1)	67 (32.3)	16 (14.7)	42 (14.7)	6 (23.1)	8 (18.2)
Daily alcohol use, n (%)	35 (27.6)	53 (25.7)	21 (19.3)	43 (15.1)	4 (18.2)	6 (14.0)
Rare alcohol use, n (%)	73 (57.5)	98 (47.6)	33 (30.3)	77 (27.0)	7 (31.8)	18 (41.9)
Information on index event						
Median duration of hospital stay (in days) at index event	3 (1–8)	4 (1–10)	4 (1–13)	5 (1–11)	3 (1–9)	3 (0–10)
Coronary event as the index event	92 (71.9)	184 (88.9)	92 (83.6)	257 (89.9)	17 (65.4)	32 (72.7)
Received coronary intervention at index event (among those with coronary event)	20 (21.7)	16 (8.7)	21 (22.8)	53 (20.6)	3 (17.6)	1 (3.1)

Median values presented with first and third quartile; n—represents the number individuals; %—is the proportion of individuals in the column; coronary artery disease is composite of myocardial infarction and coronary heart disease. Vascular and lifestyle factors measured at the start of the cohort in 1989–1990.

BMI, body mass index; BP, blood pressure; HDL, high-density lipoprotein; MACE, major adverse cardiovascular event.

### Major adverse cardiovascular event

During 4228 person-years follow-up (mean 5.3 years), 537 (67.0%) patients had a primary outcome, MACE, of which 410 (76.4%) were coronary events, 34 (6.3%) were cerebrovascular events and 93 (17.3%) were deaths due to cardiovascular disease. The median duration between the index cardiovascular event and the second MACE was 1.1 year (Q1–Q3, 0.3–4.3 years). The crude incidence rate for MACE was higher in South Asians than the other ethnic groups (117.1 per 1000 person-years in Europeans, 135.9 in South Asians and 123.6 in African Caribbeans) (table 2). However, after multivariable adjustment, the long-term risk of MACE was similar in South Asians (HR 0.97, 95% CI 0.77 to 1.21), African Caribbeans (HR 1.04, 95% CI 0.74 to 1.47) and Europeans (comparison group) (online supplemental e-figure 1). Proportional hazard assumptions were not violated (tested using Schoenfeld residuals). Focusing on non-fatal cardiovascular events, risk of recurrence in South Asians was higher than Europeans (age-adjusted and sex-adjusted HR 1.26, 95% CI 1.03 to 1.54), whereas the risk was similar in African Caribbeans and Europeans. Multivariable adjustment eliminated the excess risk in South Asians (table 2). Hypertension and diabetes were associated with a higher risk of MACE in Europeans and African Caribbeans, but not in South Asians (online supplemental e-table 3).

**Table 2:** Hazard of cardiovascular event recurrence and all-cause mortality in a tri-ethnic population in London, England between 1989 and 2017

	Total events	Person-years of follow-up	Crude rate	Unadjusted HR (95%)	Age-adjusted and sex-adjusted HR (95%)	Multivariable adjusted HR (95% CI)*
Major adverse cardiovascular event†						
European‡	207	1767	117.1	1.00	1.00	1.00
South Asian	286	2105	135.9	1.16 (0.97 to 1.39)	1.19 (0.99 to 1.43)	0.97 (0.77 to 1.21)
African Caribbean	44	356	123.6	1.04 (0.75 to 1.44)	1.06 (0.77 to 1.47)	1.04 (0.74 to 1.47)
Cardiovascular event recurrence†						
European‡	161	1767	91.1	1.00	1.00	1.00
South Asian	248	2070	119.8	1.31 (1.07 to 1.59)	1.26 (1.03 to 1.54)	1.06 (0.82 to 1.35)
African Caribbean	35	356	98.3	1.06 (0.74 to 1.53)	1.09 (0.76 to 1.58)	1.06 (0.72 to 1.56)
All-cause mortality						
European‡	159	3197	49.7	1.00	1.00	1.00
South Asian	151	4390	34.4	0.66 (0.53 to 0.83)	0.91 (0.72 to 1.14)	0.95 (0.72 to 1.26)
African Caribbean	28	583	48.0	1.01 (0.68 to 1.51)	1.07 (0.72 to 1.61)	1.07 (0.70 to 1.64)

*Multivariable adjusted model adjusted for the following: age at the index cardiovascular event, sex, vascular risk factors (known hypertension, known diabetes, body mass index, total triglycerides), lifestyle risk factors (smoking, healthy diet, physical activity and alcohol use) and days of hospitalisation at the index cardiovascular event.

†Using cause-specific proportional hazard models.

‡European ethnic group as the comparison group.

### All-cause mortality

All-cause mortality was markedly lower in South Asians compared with Europeans (unadjusted HR 0.66, 95% CI 0.53 to 0.83), which was eliminated after adjusting for age at the first cardiovascular event (HR 0.91, 95% CI 0.72 to 1.14), whereas long-term survival was not different between African Caribbeans and Europeans (HR 1.01, 95% CI 0.68 to 1.51) (table 2). The multivariable adjusted hazards of death were similar across all ethnic groups (table 2).

### Type of first event and the impact of intervention

Due to small number of index cerebrovascular events, our estimates were not precise for the subgroup with cerebrovascular disease as an index event (table 3). For coronary events, African Caribbeans were less likely to receive a coronary intervention than other ethnic groups (8.2% vs 13%) at the time of index event (online supplemental e-table 2). While receipt of coronary interventions at the time of index event was associated with a lower hazard of MACE (HR 0.63, 95% CI 0.48 to 0.83) (online supplemental e-table 4), its addition to the multivariable proportional hazards model did not alter the association between ethnicity and outcomes (table 3).

**Table 3:** Association between ethnicity and cardiovascular disease recurrence and mortality based on the nature of the index event

Type of index cardiovascular event	Coronary event	Coronary event	Cerebrovascular event
Outcomes of interest	Adjusted HR (95% CI)	Adjusted HR (95% CI)*	Adjusted HR (95% CI)
Major adverse cardiovascular event			
European	1.00	1.00	1.00
South Asian	0.94 (0.74 to 1.20)	0.95 (0.75 to 1.21)	1.08 (0.48 to 2.45)
African Caribbean	1.10 (0.74 to 1.62)	1.07 (0.72 to 1.57)	1.18 (0.43 to 3.24)
Cardiovascular event recurrence			
European	1.00	1.00	1.00
South Asian	1.00 (0.77 to 1.29)	1.01 (0.78 to 1.31)	2.46 (0.79 to 7.66)
African Caribbean	1.10 (0.72 to 1.69)	1.07 (0.70 to 1.64)	2.22 (0.57 to 8.69)
All-cause mortality			
European	1.00	1.00	1.00
South Asian	0.87 (0.64 to 1.18)	0.87 (0.64 to 1.18)	0.85 (0.38 to 1.92)
African Caribbean	1.12 (0.67 to 1.87)	1.13 (0.68 to 1.89)	0.73 (0.27 to 2.00)

*Adding receipt of coronary intervention (percutaneous coronary intervention or coronary artery bypass graft surgery) at the time of index event.

### Diabetes and time of index event

Having diabetes was associated with an increased risk of death in South Asians (HR of diabetes 1.64, 95% CI 1.15 to 2.34), but, in contrast to other ethnic groups, there was no evidence of an association between diabetes and MACE in South Asians (HR of diabetes 1.11, 95% CI 0.84 to 1.46) (online supplemental e-table 5). The interaction between South Asian ethnicity and diabetes status was of borderline significance for MACE (p interaction=0.05) but not for all-cause mortality (p interaction=0.15) (online supplemental e-table 5 and e-figure 2), and that between fasting blood glucose and ethnicity was not significant for MACE (p=0.91).

**Figure 2:**
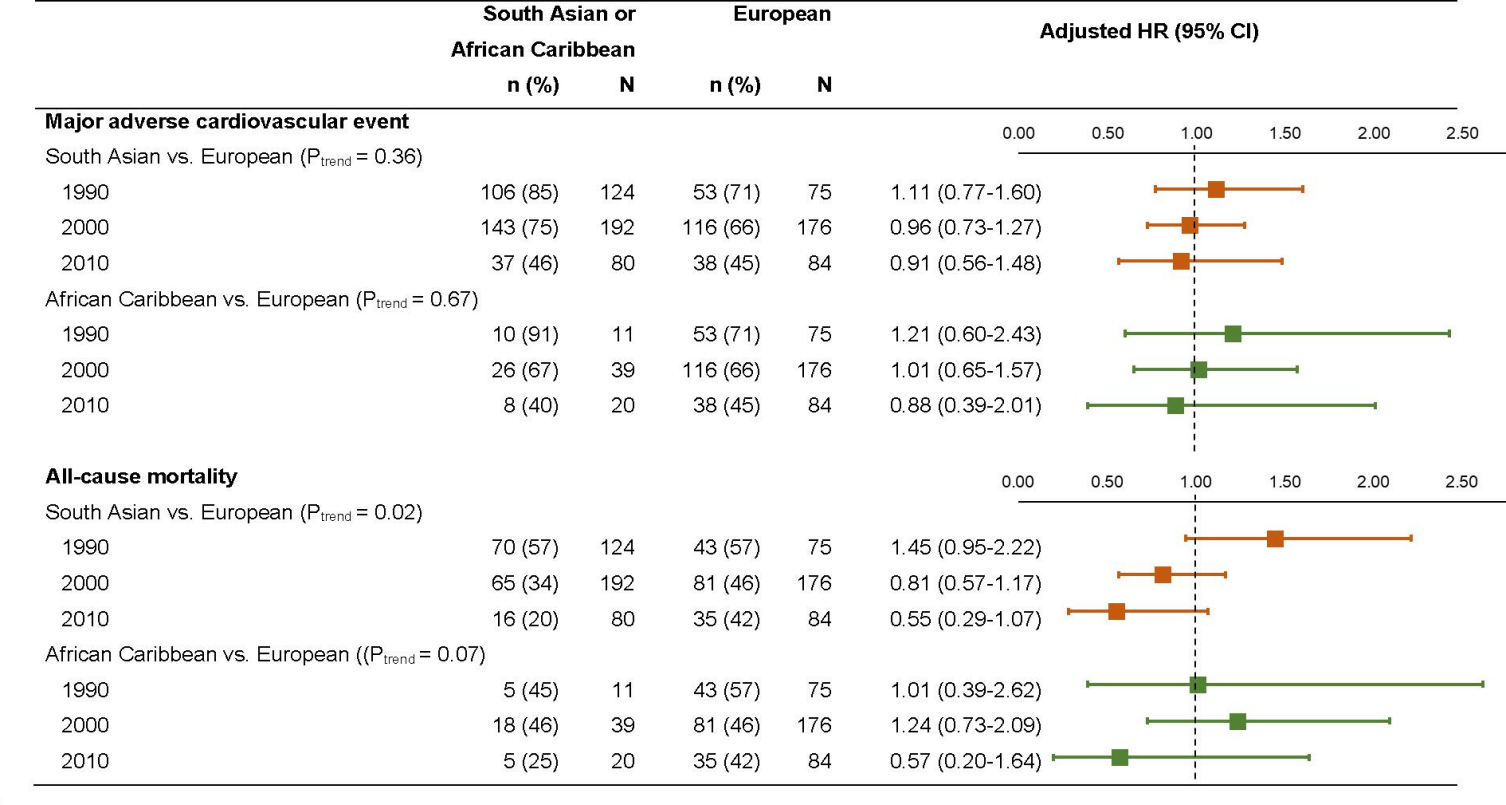
Decade of index cardiovascular event, and the association between ethnicity and major adverse cardiovascular events and all-cause mortality in a tri-ethnic cohort in London, England. P values in the parenthesis is for the interaction term (ethnicity×decade of index cardiovascular event), where ethnicity has three categories, with European as the reference group and decade of index event has three categories, with 1990 as the reference group.

There was no trend in change of the association between ethnicity and MACE based on timing of index cardiovascular event (divided into three decades: 1990, 2000, 2010), but for all-cause mortality, compared with the Europeans, there was a decline in the adjusted hazard of death based on the decade of index cardiovascular event in South Asians (p trend=0.02), and to some extent in African Caribbeans (p trend=0.07) (figure 2).

## Discussion

In a tri-ethnic cohort followed over three decades, we found that, compared with Europeans, South Asians were younger at the time of their first cardiovascular event, whereas African Caribbeans were less likely to receive coronary intervention at an index coronary event. South Asians had a 26% greater hazard of having a recurrence compared with Europeans; in large part this was due to the burden of diabetes and other cardiovascular risk factors, whereas, survival following index event was a third greater, largely accounted for by their younger at the index event compared with the Europeans. In comparison, the small sample of African Caribbeans precluded from determining differences in the risk of either outcomes between African Caribbeans and Europeans. Ethnic differences in the long-term survival following cardiovascular event declined in recent decades, whereas the evaluation of an interaction between ethnicity and diabetes was limited by small sample size.

### South Asian ethnicity

Similar to previous studies, we found that South Asians experience their index cardiovascular event at a younger age than Europeans, suggesting the need for better primary prevention in this ethnic group.^5^ A higher age-adjusted and sex-adjusted hazard of MACE and cardiovascular event recurrence in South Asians compared with other ethnic groups, suggests that secondary preventative measures are ineffective in eliminating the higher risk of cardiovascular disease in South Asians. In one study, South Asians were more likely than their white counterparts to receive secondary preventative drugs at discharge following a coronary event^14^; however, in another study the adherence to secondary preventative drugs among South Asians following cardiovascular disease was poor compared with other ethnic groups.^15^

Despite adjusting for various risk factors, reports from the SABRE cohort and a subsequent meta-analysis have found a higher risk of first coronary artery disease in South Asians compared with white subjects (HR 1.35, 95% CI 1.30 to 1.40).^14 16^ In our study, the higher risk of cardiovascular disease recurrence in South Asians compared with Europeans was eliminated after accounting for baseline cardiovascular risk factors. This could be because South Asians with baseline vascular risk factors implement better lifestyle changes, such as quitting smoking and taking up exercise, after having a cardiovascular event compared with Europeans reducing the higher risk.^17^ However, the discordant effect of South Asian ethnicity on first and recurrent event in adjusted analyses could also be due to the phenomenon called *index event bias* where risk factors for an index event are no longer relevant for recurrent events or in some cases have reversed associations.^18^ Further work will be required to study this in greater details.

Consistent with a previous study, South Asians had better long-term survival following an index cardiovascular event compared with Europeans, but in our study, this was explained by the younger age at the time of index event.^19^ It may be relevant that South Asians are at a lower risk of cancer mortality compared with Europeans, since this could also contribute to better long-term survival in South Asians.^20^ Contrary to our findings of a greater impact of diabetes on mortality among South Asians, diabetes had a smaller impact on the potential loss of life in South Asians compared with Europeans in another study.^21^ Earlier onset and longer duration of diabetes,^5^ poor glycaemic control^22^ and complications such as end-stage renal disease^23^ have been implicated for higher mortality in South Asians with diabetes compared with European counterparts.

### African Caribbean ethnicity

Similar to previous findings in England,^24^ African Caribbeans were less likely to receive coronary interventions compared with other ethnic groups in our cohort at the time of index event. We did not identify marked differences in outcomes between African Caribbeans and Europeans, although the limited number of events in African Caribbean people limited the precision of these estimates. Consistent with a previous report, African Caribbeans had the highest risk of having a cerebrovascular event as an index event,^2^ and the risk of a subsequent cerebrovascular event was higher among African Caribbeans than other ethnic groups in our study.

### Timing of index cardiovascular event

The observed reduction in the ethnic differences in mortality following an index cardiovascular event suggest that outcomes in ethnic minority groups have improved over time. Based on the design of our study, follow-up duration for someone with an index event in 1990s will be longer compared with someone with an index event in 2010s which could account for the observed differences. Changes in the severity of cardiovascular disease^25^ and better access to acute and in-hospital care and secondary prevention in ethnic minority groups^26^ may also be responsible for the observed improvement.

### Strengths and limitations

Our study is unique in following three ethnic groups from a population-based sample over a long period, combined with detailed phenotypic characterisation at recruitment. We were also able to account for receipt of intervention and duration of hospitalisation at index event, which are factors associated with our outcomes. The median duration of 1.1 years between first event and second event in our study is comparable to one,^27^ but lower than other study,^28^ suggesting variable interevent times in the literature. While we believe our findings can be generalised to South Asians migrants in the UK or other countries with equitable access to healthcare, we acknowledge that different healthcare systems may not find similar results. Due to the small proportion of African Caribbeans and women in our study, we suggest caution in interpreting our findings in people of African Caribbean ethnicity or in women. Although small, we were not able to account for missing information on some baseline risk factors listed in online supplemental appendix. We also could not include those who had a cardiovascular event for which they would not have presented to a hospital (such as a TIA or angina). We did not have information on the following factors at the time of the index hospitalisation: acute care (appropriate investigations, and possible interventions such as thrombolysis^29^), discharge outcomes (disability on discharge or location of discharge) or secondary preventative measures (adherence to medications and/or other life-style recommendations^30^), all of which would affect the risk of cardiovascular event recurrence and long-term mortality. Furthermore, risk factors included in our study as confounders could be considered mediators of the associations, and whether these were confounders, mediators or both is beyond the scope of this project, but should be considered when evaluating our findings. We also did not have information on the severity of first cardiovascular event, although we used duration of hospital stay at the time of first cardiovascular event as a proxy estimate.

### Implications of our findings

The elevated incidence of a first cardiovascular event at a young age in South Asians and the lower rate of receipt of coronary interventions in African Caribbeans should be reviewed, explanations sought and remedies implemented to address inequities. The high cumulative incidence of cardiovascular disease recurrence, especially in South Asians, suggests the need to systematically evaluate the rate and trend of cardiovascular recurrence at a population-level, and to identify measures to reduce it. Lastly, explanations for the differential changes in survival by ethnicity should be sought in order to ensure that all groups benefit optimally.

**Key messages**

**What is already known on this subject?**

- Ethnic variation in the risk of incident cardiovascular disease are known, yet little is known about ethnic variation in cardiovascular disease recurrence or long-term mortality, and how they have changed over time.

**What might this study add?**

- A higher risk of cardiovascular disease recurrence in South Asians compared with European could be explained by baseline risk vascular factors; whereas, despite lower rates of coronary intervention at the time of first cardiovascular event, there were no difference in recurrence risk between African Caribbean and European.
- There is a relative improvement in long-term survival of minority ethnic groups over time, with better survival in South Asians due to younger age at the time of index event.

**How might this impact on clinical practice?**

- Knowledge of variation in the cardiovascular disease recurrence based on ethnicity can help target secondary prevention efforts.

## Data Availability

Data are available on reasonable request. Please contact the corresponding author for data.
