# Supplementary material for: Cardiovascular disease recurrence and long-term mortality in a tri-ethnic British cohort
Source: Heart. 2020 Oct 16;107(12):996–1002. doi: 10.1136/heartjnl-2020-317641 (PMC8165149; doi:10.1136/heartjnl-2020-317641)
Supplement: Supplementary data [file heartjnl-2020-317641supp001.pdf]

APPENDIX

e-figure 1. Adjusted cumulative incidence of major adverse cardiovascular event (top), cardiovascular event recurrence (middle), and adjusted long-term survival (bottom) in a tri-ethnic cohort in London, England between 1989 and 2017, adjusted for cardiovascular risk factors.

e-Table 1. ICD-9 and ICD-10 codes for hospitalization for cardiovascular events, and OPCS codes for coronary interventions.

e-table 2. Baseline difference across three ethnic groups.

e-table 3. Results of multivariable analyses across different ethnic groups to evaluate risk factors associated with MACE in each group.

e-Table 4. Results of unadjusted and multivariable adjusted hazard ratios for major adverse cardiovascular following an index non-fatal cardiovascular event.

e-Figure 2. Distribution of major adverse cardiovascular events (composite of coronary and cerebrovascular event and cardiovascular (CV) death) in patients with diabetes and without diabetes across different ethnic groups.

e-table 5. Effect of baseline diabetes and ethnicity on major adverse cardiovascular event (MACE).

Vyas MV, *et al. Heart* 2021; 107:996–1002. doi: 10.1136/heartjnl-2020-317641

e-figure 1. Adjusted cumulative incidence of major adverse cardiovascular event (top), cardiovascular event recurrence (middle), and adjusted long-term survival (bottom) in a tri-ethnic cohort in London, England between 1989 and 2017, adjusted for cardiovascular risk factors.

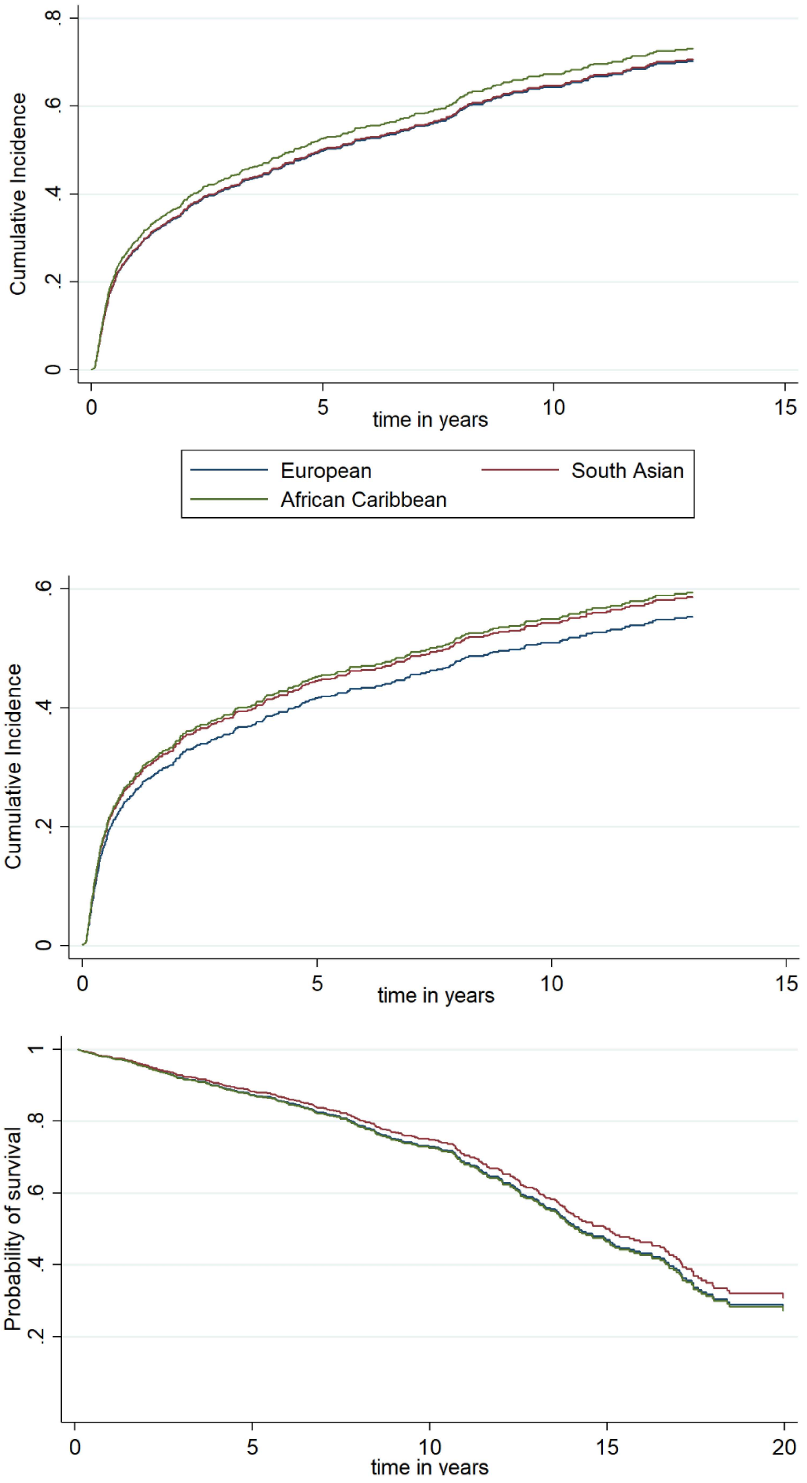

Vyas MV, *et al. Heart* 2021; 107:996–1002. doi: 10.1136/heartjnl-2020-317641

e-Table 1. ICD-9 and ICD-10 codes for hospitalization for cardiovascular events, and OPCS codes for coronary interventions.

**Cardiovascular disease sub-type**	**ICD-9 codes**	**ICD-10 codes**
Ischemic heart disease	412 413 414	I20 I24 I23
Myocardial infarction	410 411	I21 I122
Stroke or transient ischaemic attack	430 431 434 436<br>3623	I60 I61 I63 I64<br>H340 H341 G450 G451 G452 G453 G458 G459

**Codes in the OPCS Classification of Surgical Operations and Procedures**	**Type of intervention**
K40	Saphenous vein graft replacement of coronary artery
K41	Other autograft replacement of coronary artery
K42	Allograft replacement of coronary artery
K43	Prosthetic replacement of coronary artery
K44	Other replacement of coronary artery
K45	Connection of thoracic artery to coronary artery
K46	Other bypass of coronary artery
K49	Transluminal balloon angioplasty of coronary artery
K50	Percutaneous transluminal coronary angioplasty
K75	Percutaneous transluminal balloon angioplasty and stenting of coronary

OPCS – Office of Population Censuses and Surveys, ICD – International Classification of Diseases

e-table 2. Baseline difference across three ethnic groups.

	European (n = 335)	South Asian (n = 396)	African Caribbean (n = 70)
Demographic information			
Median age (years) at index event	69 (64-75)	66 (59-71)	70 (63-74)
Female, n (%)	61 (18.2)	60 (15.2)	27 (38.6)
Median years of education	10 (9-11)	12 (10-14)	10 (9-11)
Vascular risk factors			
Known diabetes, n (%)	27 (8.0)	106 (26.8)	19 (27.1)
Known hypertension, n (%)	30 (9.0)	59 (14.9)	20 (28.6)
Median fasting blood glucose (mmol/L)	5.4 (5.1-5.8)	5.4 (5.2-6.4)	6.0 (5.3-6.5)
Median total cholesterol (mmol/L)	6.3 (5.6-7.0)	6.0 (5.3-6.7)	5.7 (5.0-6.6)
Median HDL (mmol/L)	1.3 (1.1-1.5)	1.2 (1.0-1.3)	1.5 (1.2-1.8)
Median triglycerides (mmol/L)	1.6 (1.1-2.1)	1.8 (1.3-2.6)	1.2 (0.9-1.6)
Median systolic BP (mmHg)	124 (114-135)	125 (114-137)	130 (120-138)
Median diastolic BP (mmHg)	79 (72-86)	80 (74-87)	82 (75-88)
Median BMI (kg/m^2)	26.3 (23.8-28.8)	26.1 (24.2-28.2)	27.5 (25.1-29.8)
Median waist-hip ratio	0.9 (0.9-1.0)	1.0 (0.9-1.0)	0.9 (0.9-1.0)
Lifestyle factors			
Physically active, n (%)	193 (57.6)	169 (42.7)	35 (50.0)
Healthy diet, n (%)	117 (34.9)	142 (36.3)	28 (40.0)
Ex-smoker, n (%)	121 (36.1)	31 (7.9)	7 (10.0)
Current smoker, n (%)	103 (30.8)	58 (14.7)	14 (20.0)
Daily alcohol consumption, n (%)	88 (26.4)	64 (16.2)	10 (15.4)
Rare alcohol consumption, n (%)	171 (51.4)	110 (27.9)	25 (38.5)
Information on index event			
Median duration of hospital stay (in days) at index event	4 (1-9)	4 (1-10)	2 (1-7)
Coronary event as the index event	276 (82.4)	349 (88.1)	49 (70.0)
Received coronary intervention at index event if it was a coronary event	36 (13.0)	74 (13.5)	4 (8.2)

Median values presented with 1st and 3rd quartile; n – represents the number individuals; % - is the proportion of individuals in ethnic group.
Total cholesterol, HDL, Triglycerides, and blood glucose are fasting measures in serum measured as mmol/L
Coronary event is a composite of non-fatal myocardial infarction or ischaemic heart disease.
Abbreviations: HDL – high density lipoprotein, BMI – body mass index, BP – blood pressure.

Vyas MV, *et al. Heart* 2021; 107:996–1002. doi: 10.1136/heartjnl-2020-317641

e-table 3. Results of multivariable analyses across different ethnic groups to evaluate ~~predictors~~ risk factors associated with ~~of~~ MACE in each group.

	European	P value	South Asians	P value	African Caribbean	P value
Number of events/Total number of individuals	204/330		281/390		42/64	
**Variables of interest**						
Female	1.02 (1.00-1.04)	0.20	1.01 (0.99-1.02)	0.28	0.98 (0.93-1.03)	0.87
Age at index event	0.81 (0.54-1.21)	0.11	0.81 (0.55-1.19)	0.47	0.93 (0.39-2.24)	0.48
Diabetes	1.90 (1.19-3.03)	**<0.01**	1.14 (0.85-1.51)	0.39	2.16 (0.86-5.45)	0.10
Hypertension	1.67 (1.04-2.67)	**0.03**	0.86 (0.61-1.21)	0.37	1.11 (0.48-2.55)	0.81
Triglyceride levels	1.10 (0.98-1.23)	0.11	1.10 (1.03-1.17)	**<0.01**	0.77 (0.41-1.44)	0.41
Body mass index	0.98 (0.95-1.02)	0.37	0.98 (0.94-1.01)	0.16	1.05 (0.95-1.15)	0.36
Smoking status		0.35		0.86		0.22
Current vs. never smoker	1.25 (0.87-1.80)		0.99 (0.69-1.40)		0.44 (0.17-1.12)	
Ex-smoker vs. never smoker	0.99 (0.69-1.43)		0.88 (0.56-1.39)		0.74 (0.25-2.20)	
Alcohol consumption		0.17		0.36		0.47
Daily vs. never	0.71 (0.47-1.07)		0.82 (0.57-1.17)		1.04 (0.38-2.84)	
Rare vs. never	0.73 (0.51-1.05)		0.84 (0.63-1.12)		1.70 (0.68-4.26)	
Healthy Diet	0.81 (0.60-1.10)	0.19	0.91 (0.71-1.18)	0.48	0.78 (0.33-1.81)	0.56
Days of hospital stay	1.00 (0.99-1.00)	0.30	0.99 (0.98-1.00)	0.13	1.00 (0.99-1.02)	0.71

Vyas MV, *et al. Heart* 2021; 107:996–1002. doi: 10.1136/heartjnl-2020-317641

e-Table 4. Results of unadjusted and multivariable adjusted hazard ratios for major adverse cardiovascular following an index non-fatal cardiovascular event.

**Outcome of interest**		**MACE**	
**Covariates**	Effective sample size	Unadjusted HRs	Full-adjusted HRs^
Age at first CVD	801	1.01 (1.00-1.02)	1.01 (1.00-1.02)
Female	801	0.88 (0.70-1.10)	0.84 (0.65-1.08)
Years of education	759	0.99 (0.96-1.01)	**-**
Deprivation index	695	0.90 (0.68-1.19)	-
Known diabetes	**801**	**1.45 (1.18-1.78)**	**1.31 (1.04-1.65)**
Fasting blood sugar	**796**	**1.05 (1.01-1.08)**	-
Known hypertension	800	1.12 (0.88-1.42)	1.05 (0.81-1.35)
systolic BP	800	1.00 (1.00-1.01)	-
diastolic BP	800	1.00 (0.99-1.01)	-
Total cholesterol	795	1.05 (0.97-1.13)	-
HDL	**778**	**0.64 (0.50-0.82)**	-
Triglycerides	**795**	**1.09 (1.04-1.15)**	**1.09 (1.03-1.15)**
Body mass index	801	0.99 (0.97-1.01)	0.98 (0.96-1.01)
Waist-hip ratio	801	2.17 (0.84-5.60)	-
Smoking status			
Current vs. never smoker	799	0.79 (0.79-1.20)	1.01 (0.80-1.27)
Ex-smoker vs. never smoker	799	0.88 (0.71-1.10)	0.89 (0.69-1.15)
Alcohol consumption			
Daily vs. never	792	0.80 (0.63-1.00)	0.77 (0.60-0.99)
Rare vs. never	792	**0.82 (0.68-0.99)**	0.81 (0.66-1.00)
Physically active*	801	1.03 (0.87-1.22)	-
Healthy diet	796	0.86 (0.72-1.03)	0.87 (0.72-1.05)
Days of hospitalisation at index event	801	1.00 (1.00-1.01)	1.00 (0.99-1.00)
For subgroup of patients who had coronary event as the index cardiovascular event			
Received intervention if first event was Coronary	674	**0.69 (0.53-0.89)**	**0.63 (0.48-0.83)**

^full adjusted model adjusted for ethnicity in addition to those listed in column, and excluded those covariates that were collinear to the ones retained in the final model (e.g., SBP and DBP vs. known hypertension).
Healthy diet was defined as having fruit and vegetables daily; *physically active* was defined as an individual with a score on physical activity scale over median value.
*Because it did not meet proportional hazards assumption, it was treated as a *strata* variable in the model, and so has no coefficient.

e-Figure 2. Distribution of major adverse cardiovascular events (composite of coronary and cerebrovascular event and cardiovascular (CV) death) in patients with diabetes and without diabetes across different ethnic groups.

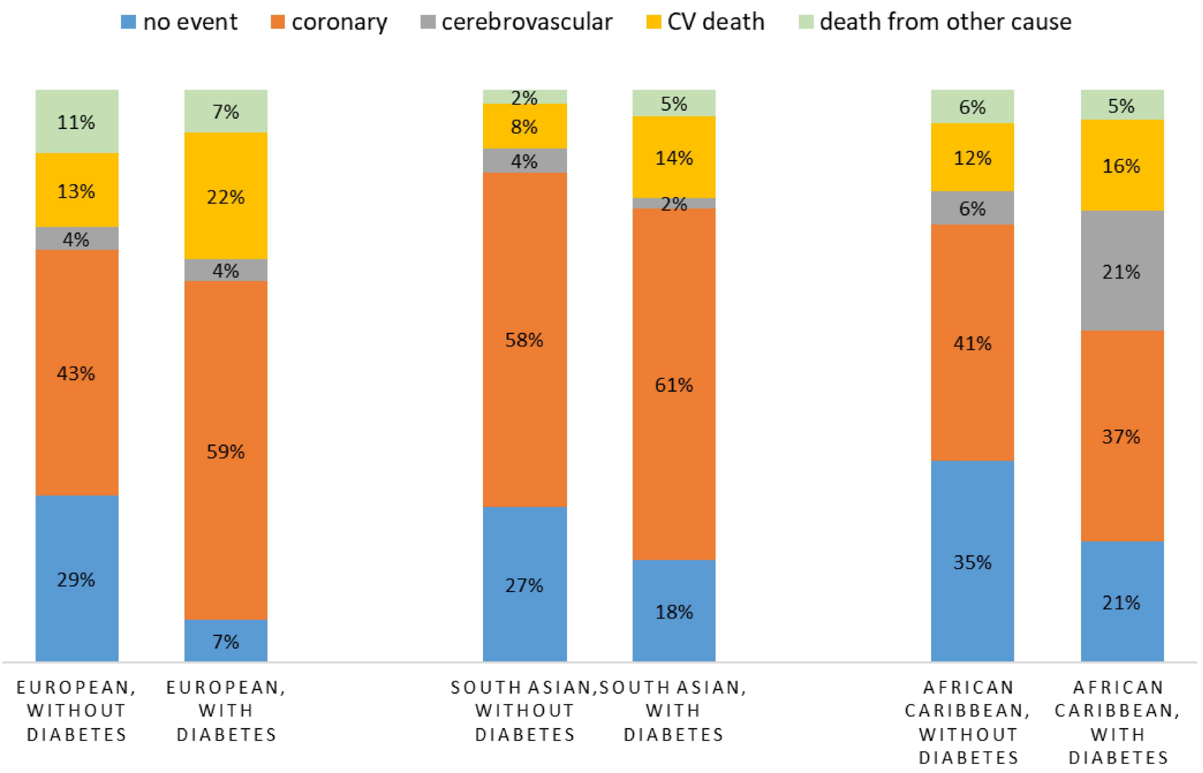

e-table 5. Effect of baseline diabetes and ethnicity on major adverse cardiovascular event (MACE).

	**Patients with diabetes**		**Patients without diabetes**		**Adjusted HR (95% CI)†**
	**# events (%)**	**total**	**# events (%)**	**total**	**diabetes vs. no diabetes**
Major adverse cardiovascular event*					
European	23 (85.2)	27	184 (59.7)	308	1.85 (1.18-2.89)
South Asian ***(P = 0.05)***	82 (77.4)	106	204 (70.3)	290	1.11 (0.84-1.46)
African Caribbean ***(P = 0.85)***	14 (73.7)	19	30 (58.8)	51	1.99 (1.02-3.89)
All-cause mortality					
European	18 (66.7)	27	141 (45.8)	308	1.01 (0.58-1.78)
South Asian ***(P = 0.15)***	55 (51.9)	106	96 (33.1)	290	1.64 (1.15-2.34)
African Caribbean ***(P = 0.19)***	10 (52.6)	19	18 (35.3)	51	1.94 (0.86-4.36)

*using cause-specific proportional hazard models; P values in the parenthesis are for the interaction between ethnicity, (South Asian or African Caribbean, with European as the reference group) and diabetes status (no diabetes as the reference group).
†Multivariable adjusted model adjusted for the following: age at the index cardiovascular event, sex, cardiovascular risk factors (known hypertension, body mass index, and total triglycerides), lifestyle risk factors (smoking, healthy diet, physical activity, and alcohol use), and days of hospitalisation at the index cardiovascular event.
Abbreviations: HR – hazard ratios, CI – confidence interval.
